# Supplementary figures and images for: Predictors of Discordance in the Assessment of Skeletal Muscle Mass between Computed Tomography and Bioimpedance Analysis
Source: J Clin Med. 2019 Mar 7;8(3):322. doi: 10.3390/jcm8030322 (PMC6462955; doi:10.3390/jcm8030322)

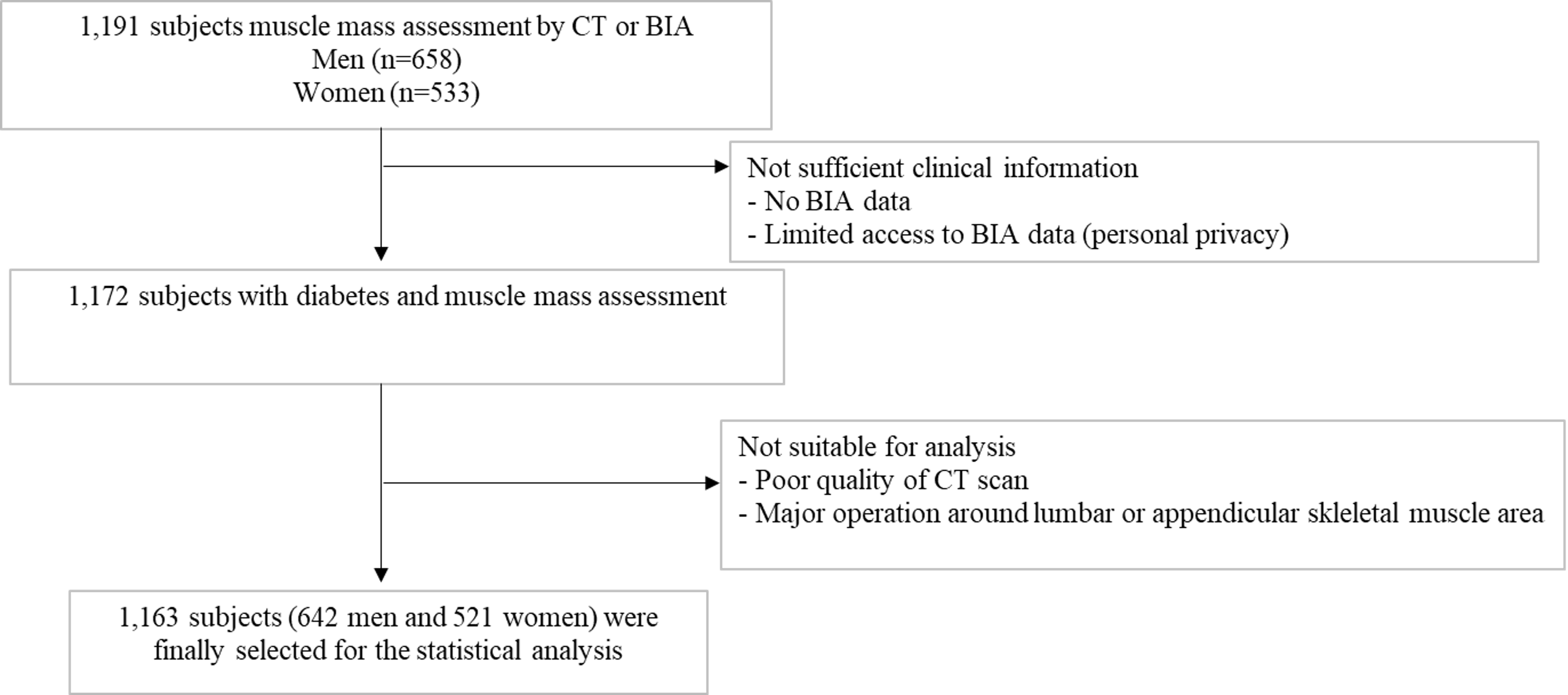

Supplement: Supplementary file 1 [file jcm-08-00322-s001.zip › supfigure1.tif]

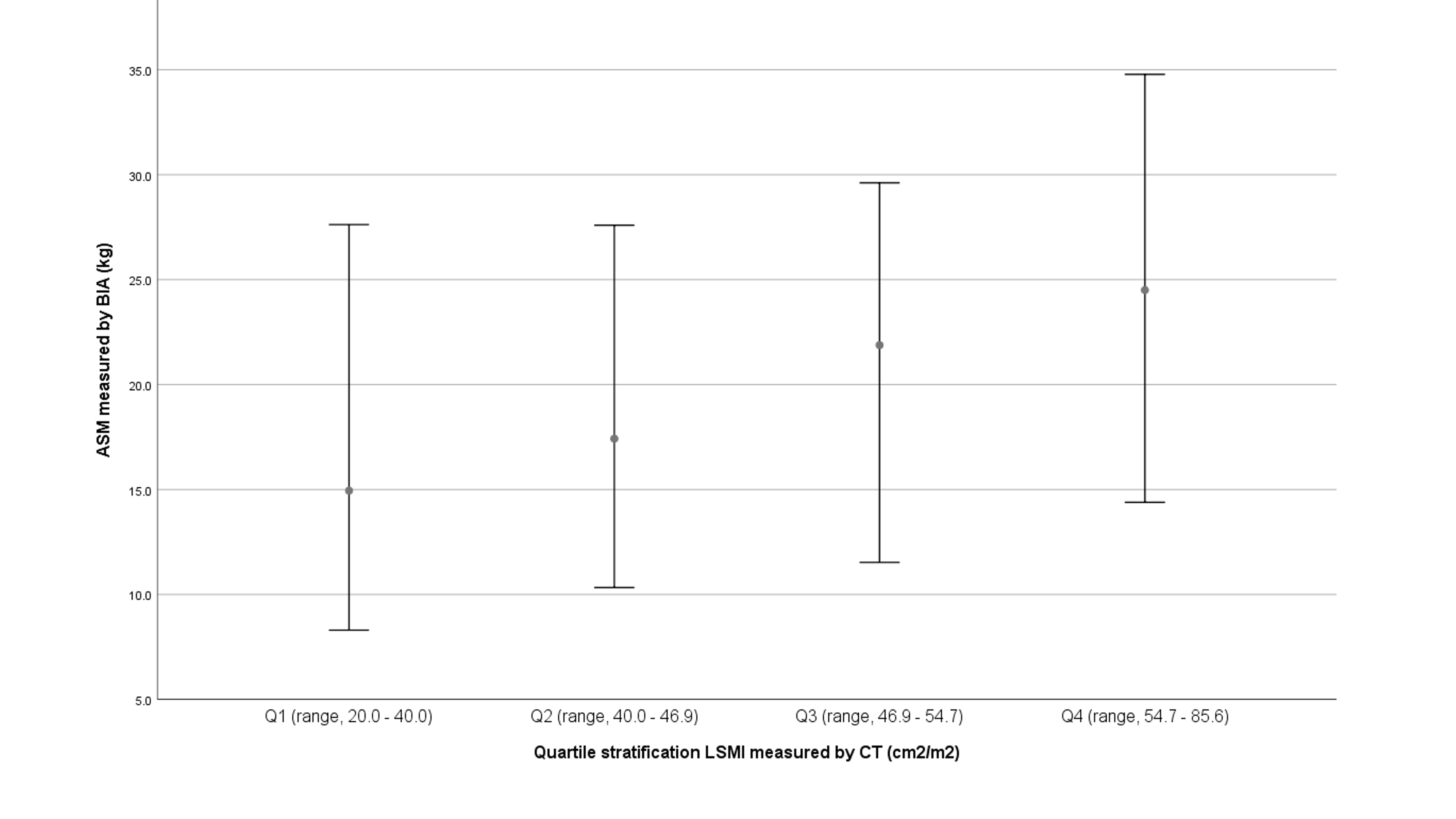

Supplement: Supplementary file 1 [file jcm-08-00322-s001.zip › supfigure2.tif]

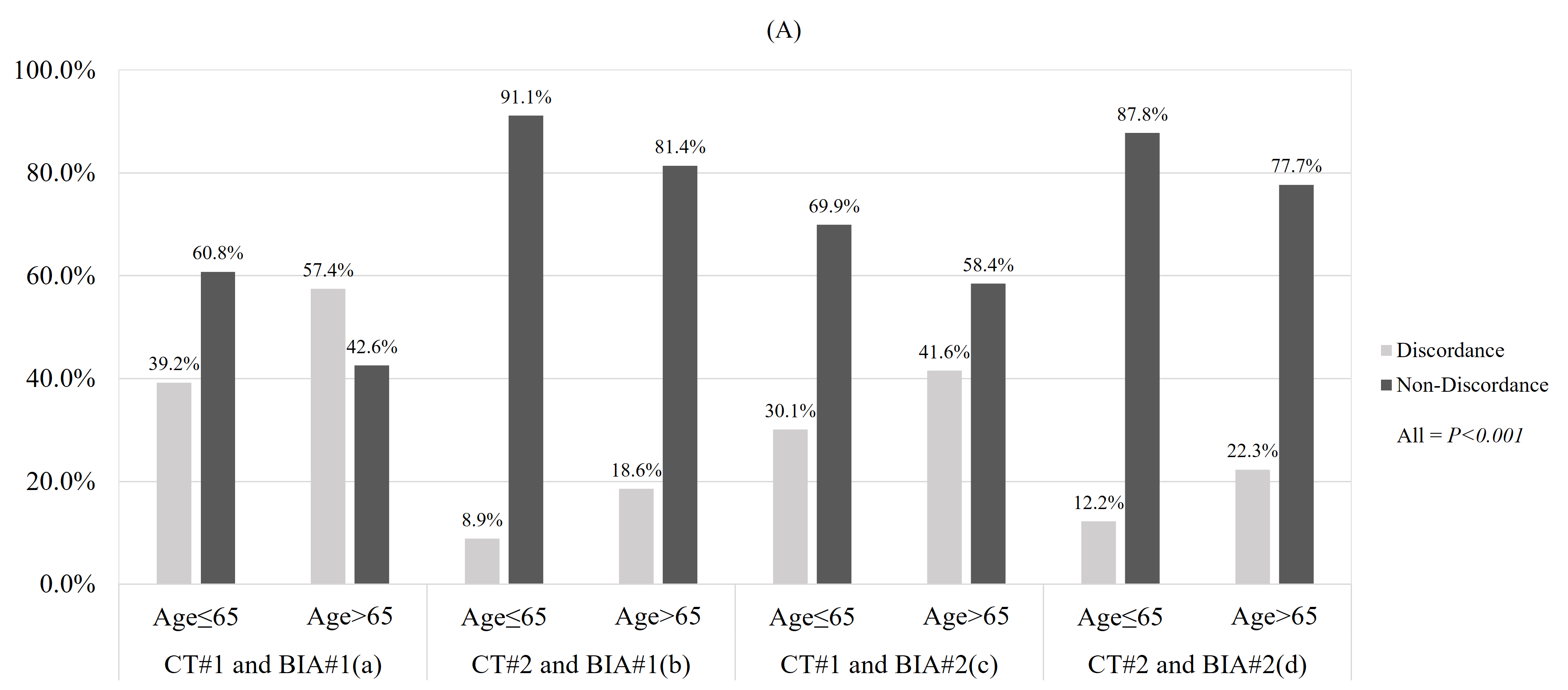

Supplement: Supplementary file 1 [file jcm-08-00322-s001.zip › supfigure3A.TIF]

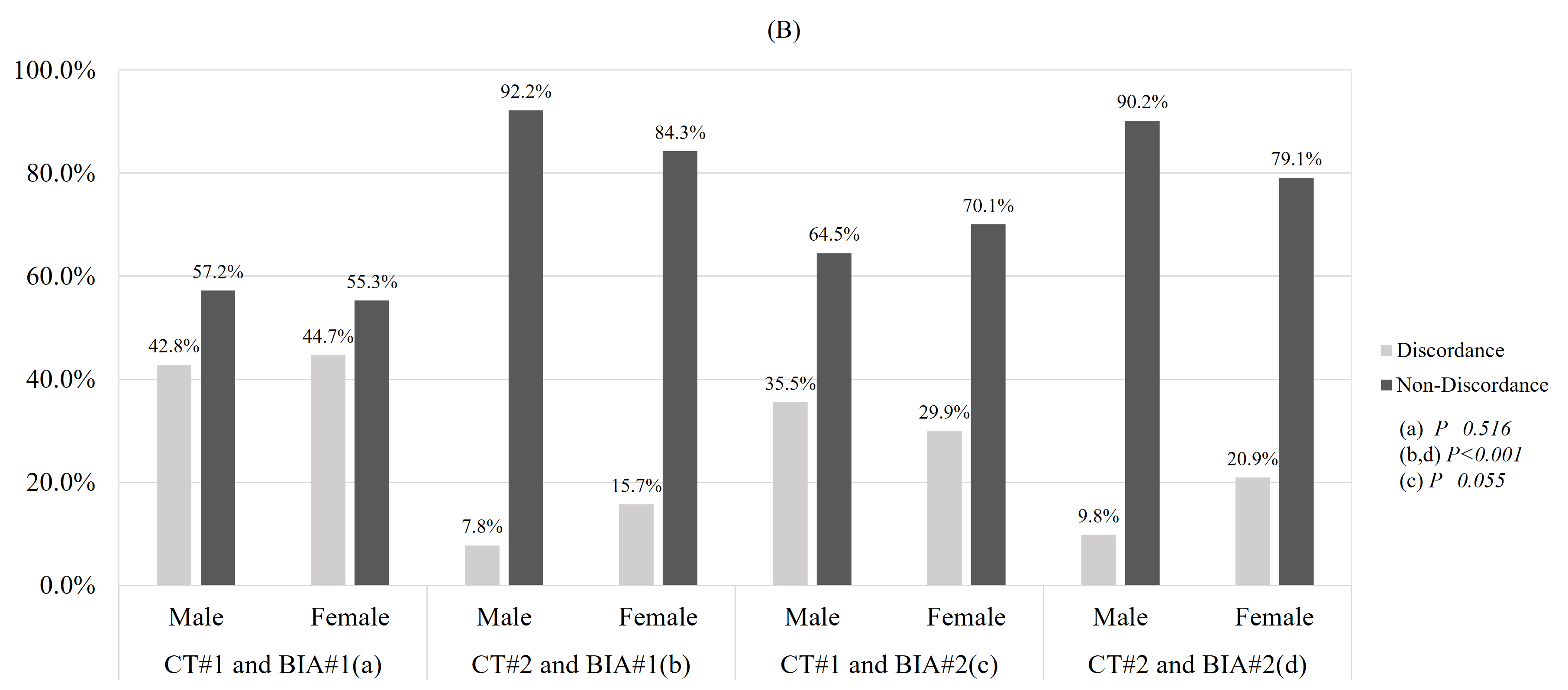

Supplement: Supplementary file 1 [file jcm-08-00322-s001.zip › supfigure3B.TIF]

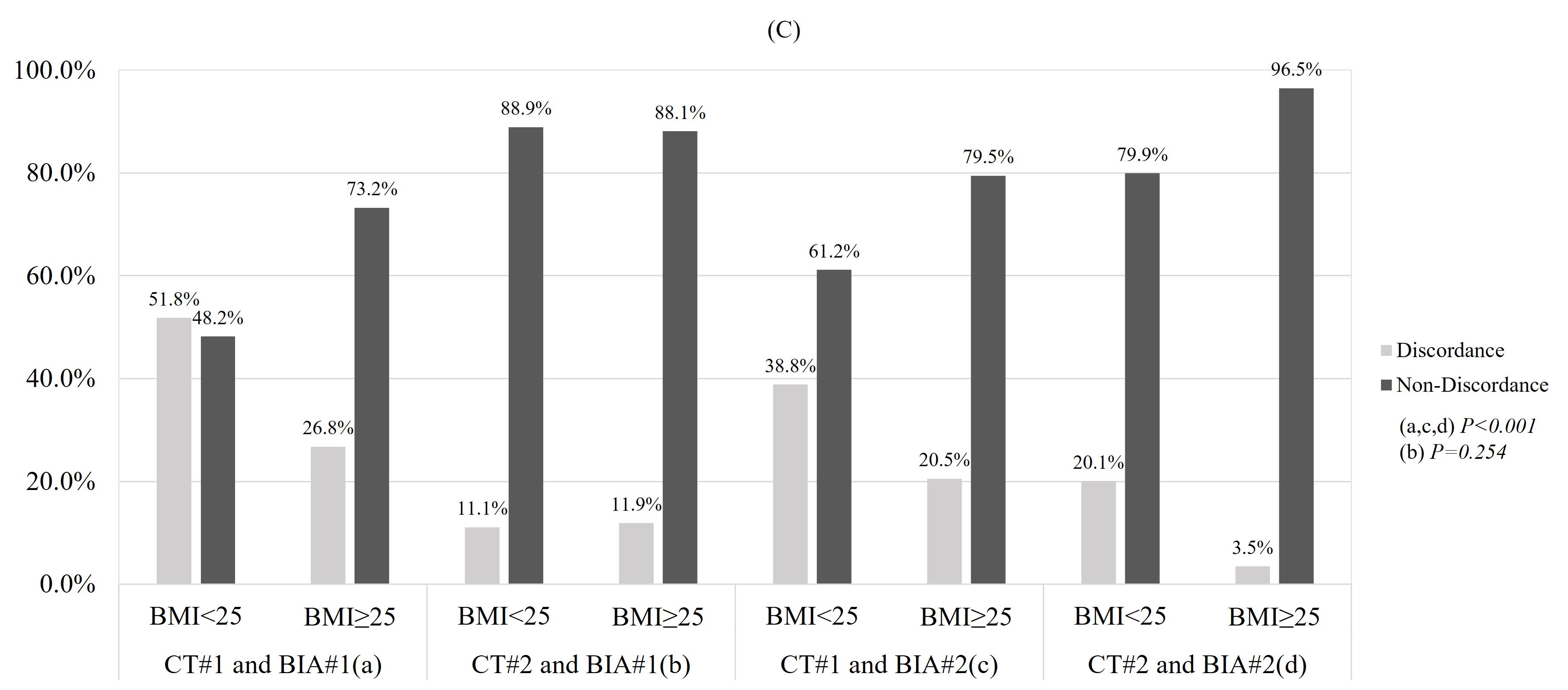

Supplement: Supplementary file 1 [file jcm-08-00322-s001.zip › supfigure3C.TIF]

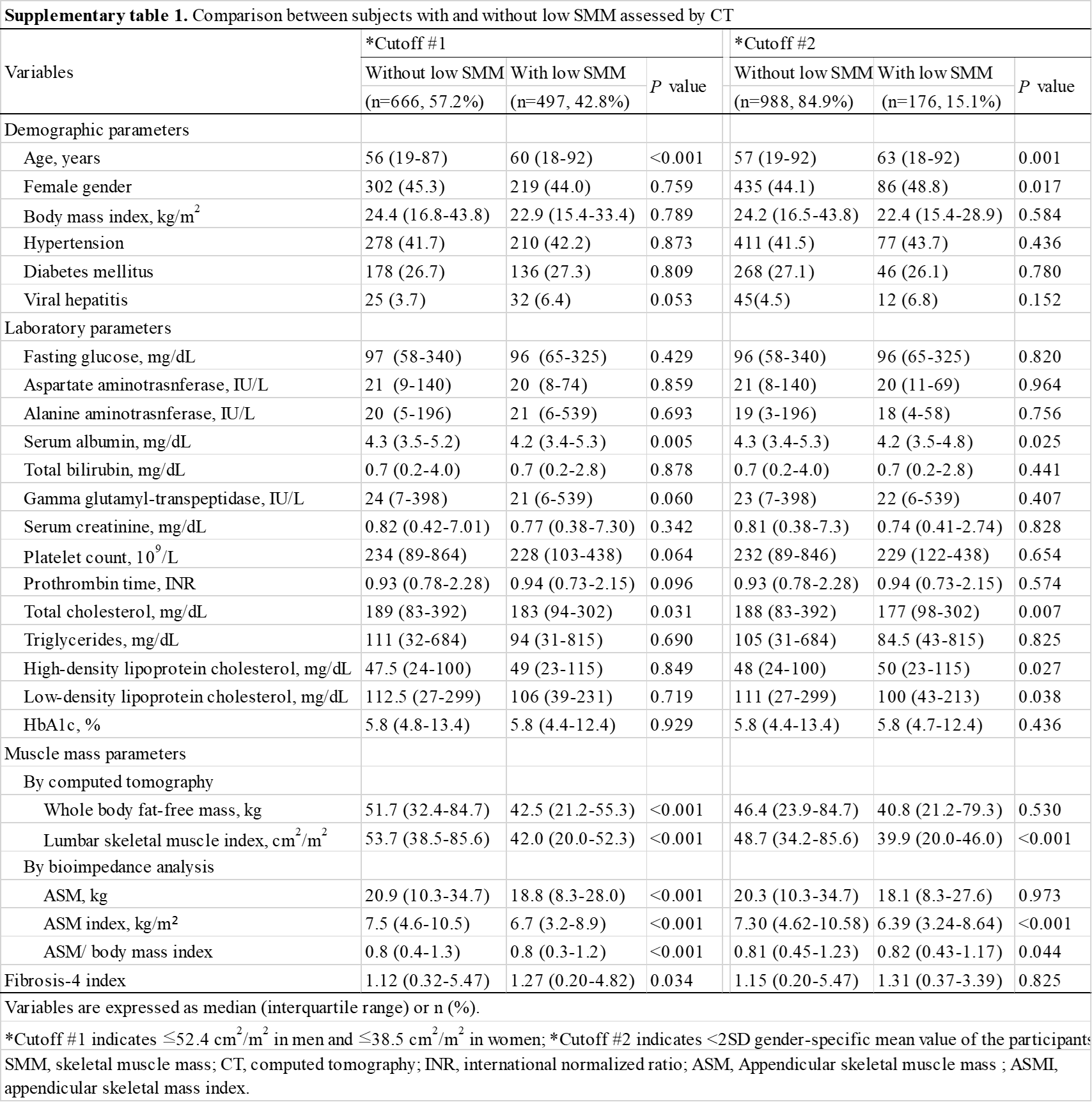

Supplement: Supplementary file 1 [file jcm-08-00322-s001.zip › Suptable1.tif]

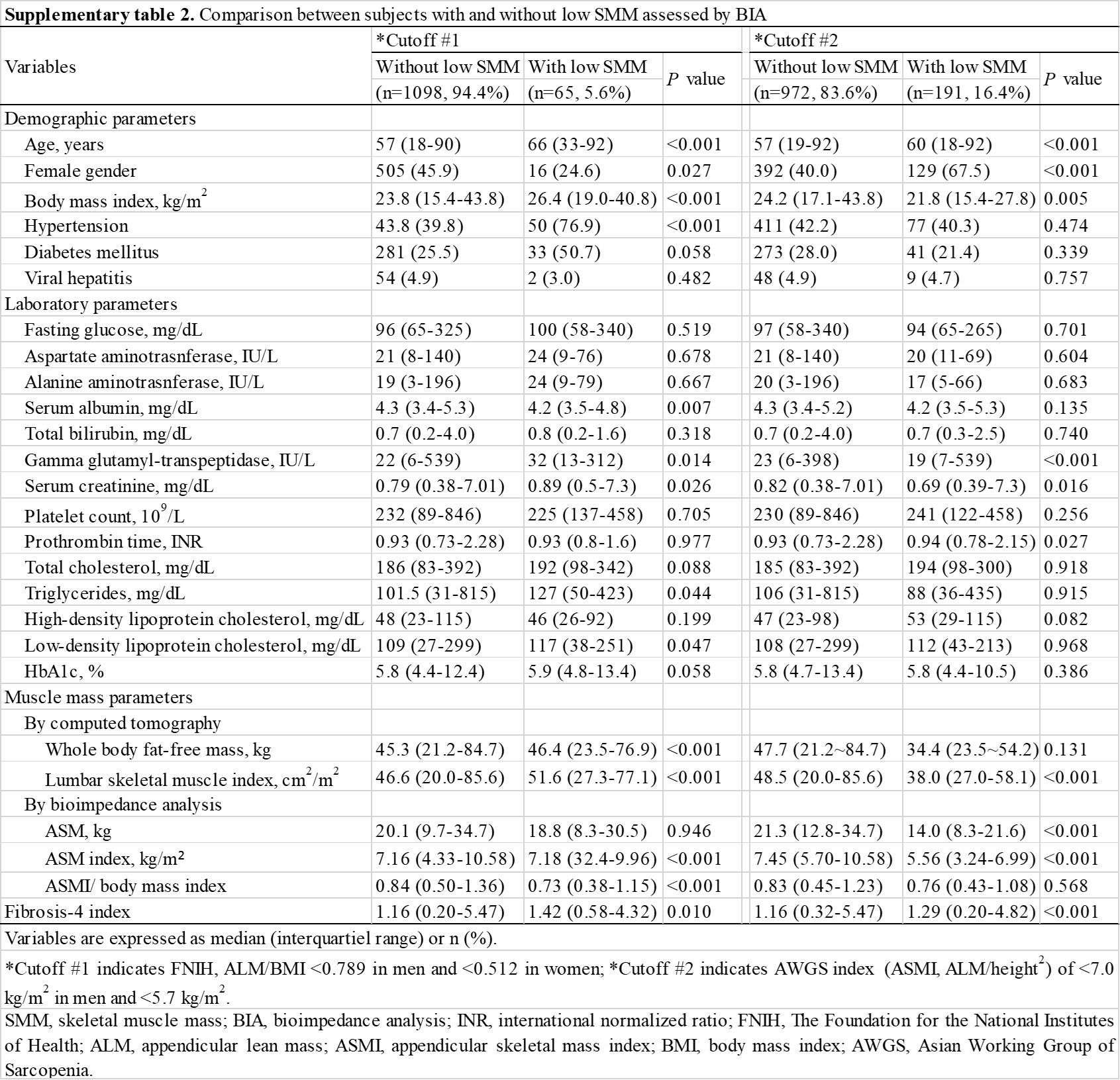

Supplement: Supplementary file 1 [file jcm-08-00322-s001.zip › Suptable2.tif]

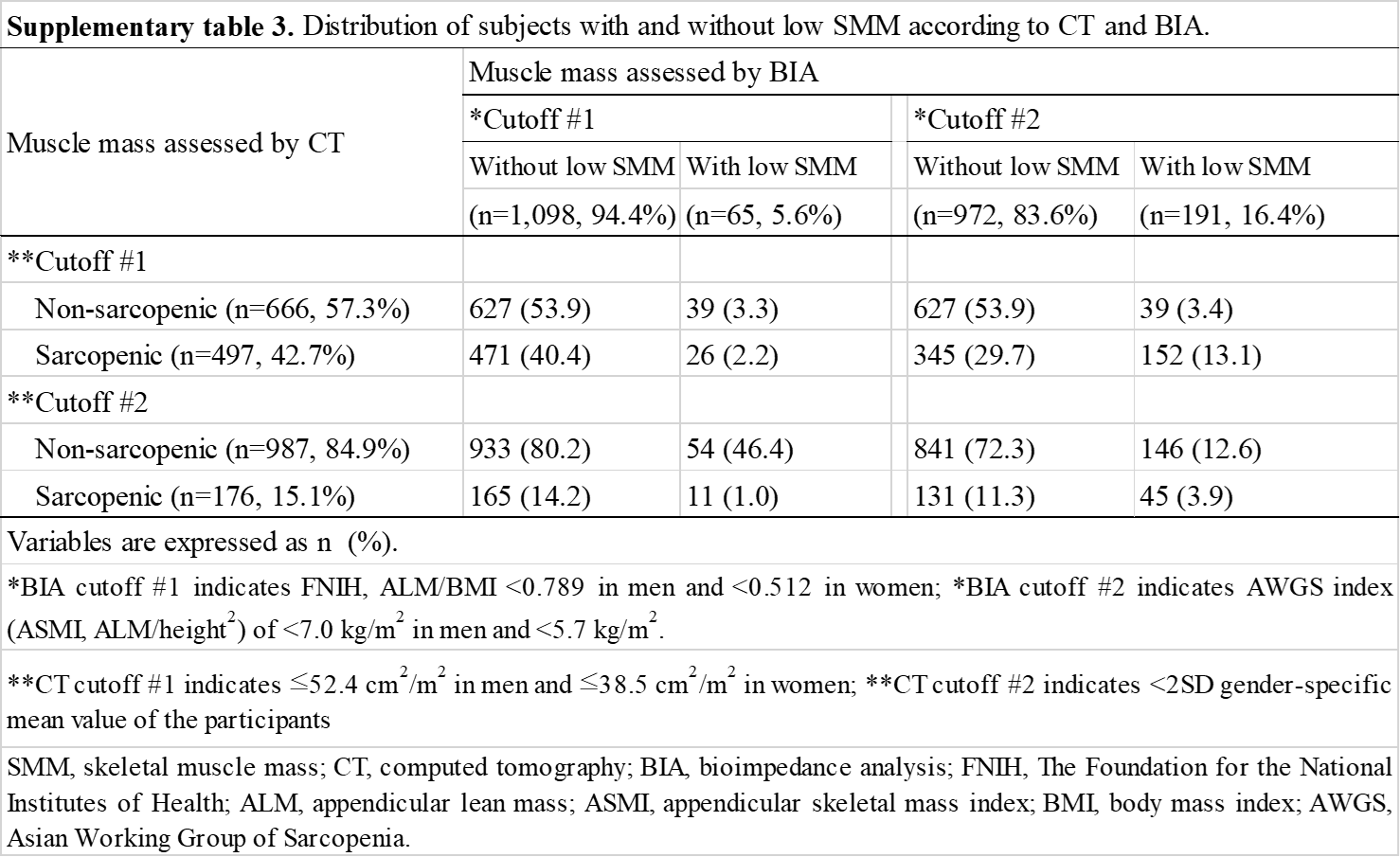

Supplement: Supplementary file 1 [file jcm-08-00322-s001.zip › Suptable3.tif]

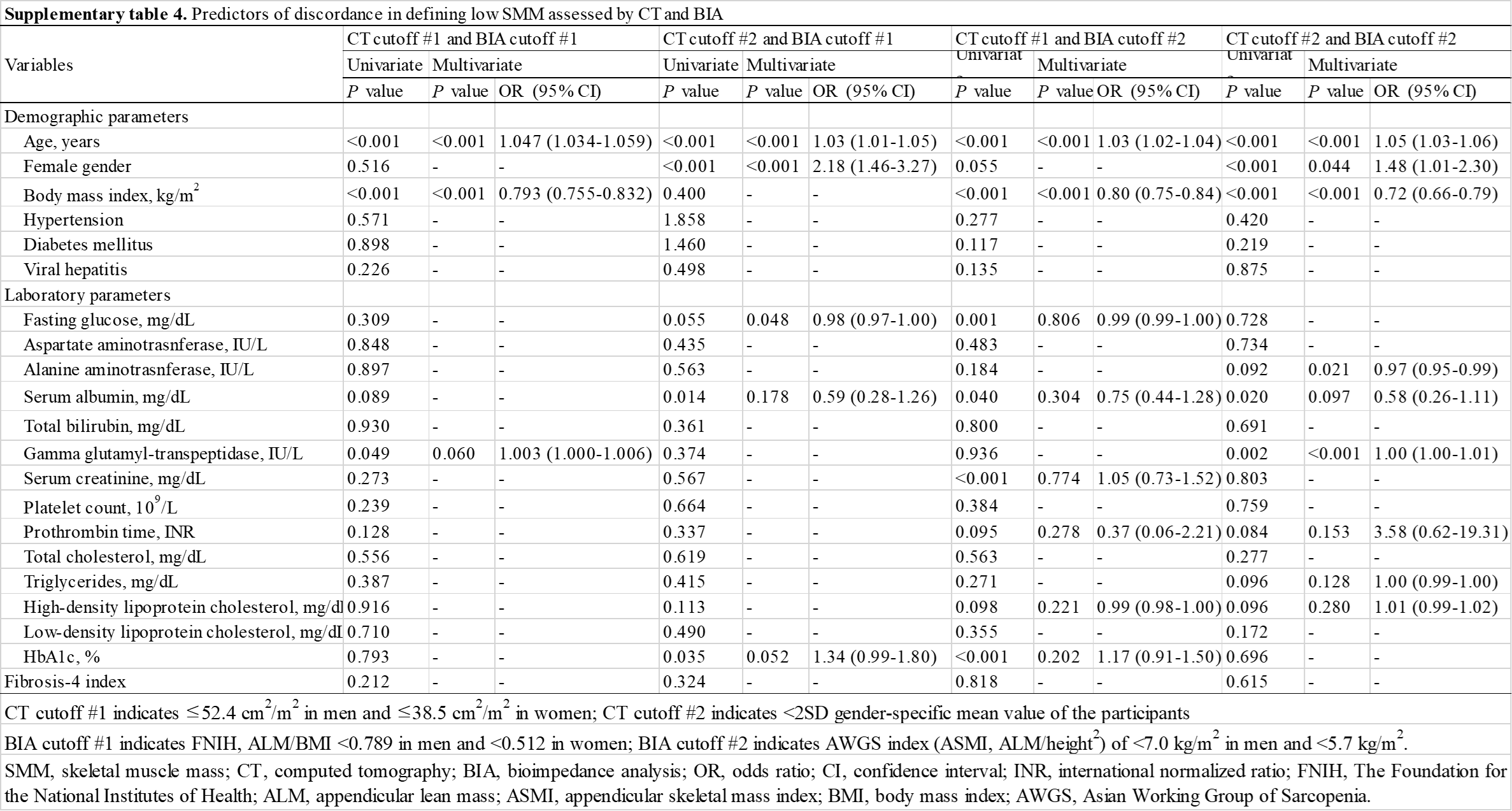

Supplement: Supplementary file 1 [file jcm-08-00322-s001.zip › Suptable4.tif]

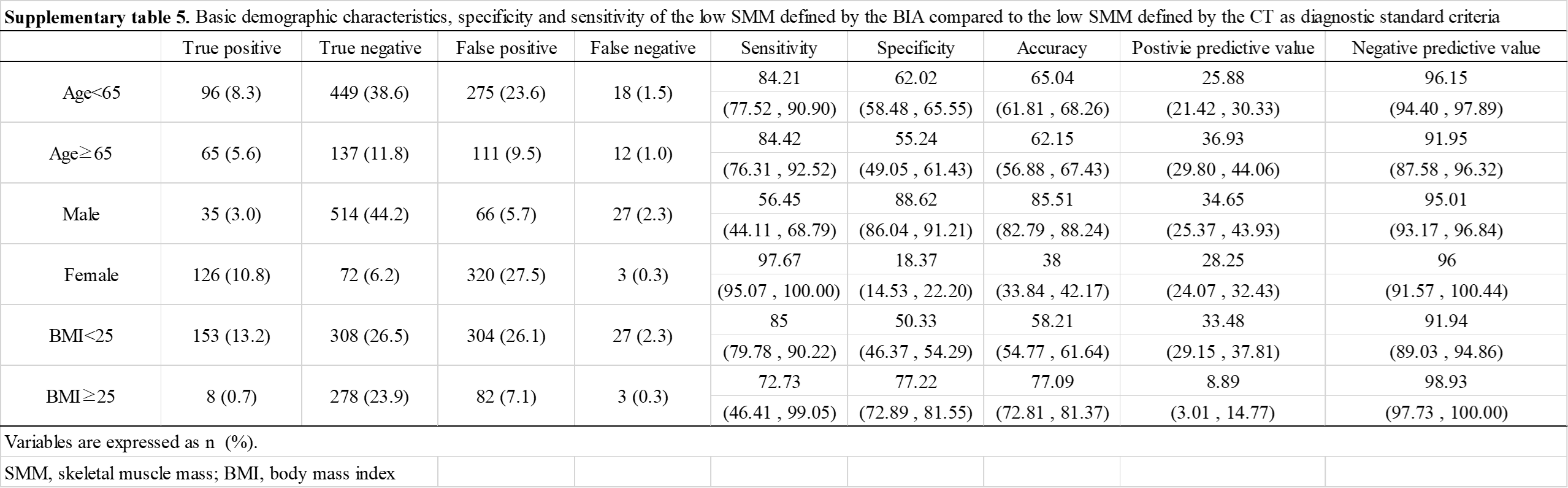

Supplement: Supplementary file 1 [file jcm-08-00322-s001.zip › Suptable5.tif]

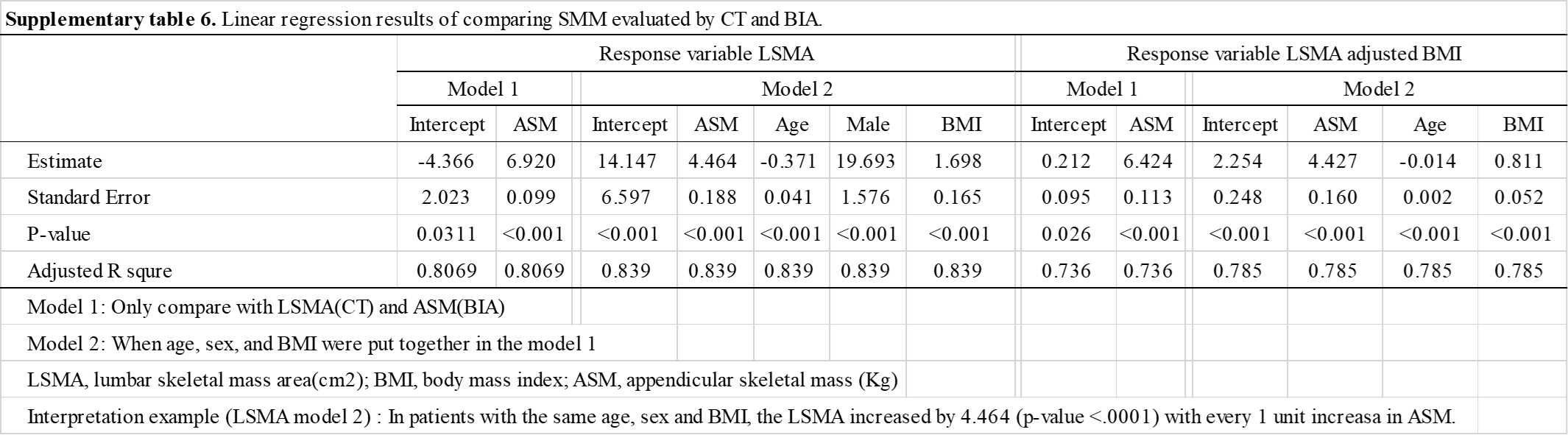

Supplement: Supplementary file 1 [file jcm-08-00322-s001.zip › Suptable6.tif]
